# Artificial Weathering Test Methods of Waterborne Acrylic Coatings for Steel Structure Corrosion Protection

**DOI:** 10.3390/ma17081857

**Published:** 2024-04-17

**Authors:** Łukasz Ładosz, Ewa Sudoł, Ewelina Kozikowska, Emilia Choińska

**Affiliations:** 1 Construction Materials Engineering Department, Instytut Techniki Budowlanej, 00-611 Warsaw, Poland; e.sudol@itb.pl (E.S.); e.kozikowska@itb.pl (E.K.); 2 Faculty of Materials Science and Engineering, Warsaw University of Technology, 02-507 Warsaw, Poland; emilia.choinska@pw.edu.pl

**Keywords:** tests methods, weathering, artificial tests, eco-friendly, waterborne coatings, original cyclic test, change of properties

## Abstract

Corrosion protection technologies based on waterborne paints have become increasingly popular as steel structure protection, which implies the need to determine relevant assessment methods considering the conditions of use and product-specific characteristics. This study attempts to evaluate the fitness of standard corrosion protection weathering methods and an original cyclic test for verifying the resistance of waterborne acrylic coatings to environmental conditions. Changes to the properties of artificially weathered coatings were analysed with reference to those observed during exposure in natural conditions. The degree of coating degradation after exposure to neutral salt spray and condensation humidity was determined to significantly exceed the changes observed in natural conditions. An original cyclic test caused changes in the appearance, microstructure, FT-IR spectrum and utility properties of the coatings, such as thickness, colour, hardness, adhesion and impedance, similar to those observed in the natural environment. The results confirm that the programming direction of waterborne coatings artificial weathering tests is adequate and promising.

## 1. Introduction

Despite many advantages, structural steel is susceptible to corrosion in most environments. Corrosion is estimated to be responsible for losses amounting to 3–4% of the global gross domestic product [1,2]. Owing to adequate corrosion protection, the costs can be reduced by up to 35%, but, most importantly, the risk of construction disasters can be limited [3]. Steel corrosion protection involves applying organic coatings as well as using inhibitors or electrochemical protection [4]. Organic coatings are the most popular. This study focuses on eco-friendly waterborne organic coatings. They are not as popular as solvent-based coatings in construction but have become increasingly widespread due to limitations in the use of pigmented paints, e.g., containing lead tetroxide or chromium compounds with oxidation state +6 [3,5].

The main advantage of waterborne coatings compared to solvent-based ones is an up to 95% lower content of volatile organic compounds (VOCs) and lower price [3]. Still, they are less prevalent than solvent-based coatings because of their higher application regime. The surface needs to be better prepared. Nevertheless, the prognosis is promising, as through the polymerization of monomers in aggregated form, improvements in wettability have been achieved [6]. Environmental conditions should be observed more strictly during application as well. They are less efficient, have limited coating thickness, and have much longer drying times [3,4].

The results of previous studies revealed that waterborne coatings are less durable than solvent-based ones [7,8,9]. Nevertheless, eco-friendly waterborne systems have developed dynamically in striving for sustainable development. Waterborne and durable solid slippery and superhydrophobic coatings, resistant to abrasion, have been developed [10]. Coating manufacturers aim to achieve solutions with utility properties that are at least on par with those of solvent-based coatings [5,11]. The changes in the corrosion protection sector, resulting in innovative waterborne paints, entail improving research methods to consider the products’ specificity [12]. 

An analysis of the literature data revealed that the currently used accelerated weathering methods, indispensable to verify the durability of corrosion protection systems, were designed for standard solvent-based paints. For different compositions of organic solvent-based and waterborne products, the previous accelerated weathering methods may be inadequate. Weathering tests are typically based on intensive moisture impact, often, to an extent, exceeding natural impacts [13,14], which may lead to misevaluation, considering the sensitivity of waterborne paints to water.

In natural conditions, coatings are exposed to many environmental factors of varied individual intensity. The main degradation factors for polymer coatings include temperature, humidity, solar radiation, wind, atmospheric precipitation, and air pollution (SO_2_, NOx, and O_3_) [8,15,16,17,18,19,20]. Simulating the impacts in laboratory conditions is possible but with numerous technical limitations for their combination. This might result in a lack of correlation between accelerated test results and natural effects [7]. Davalos-Monterio et al. [21] demonstrated, by examining powder coating corrosion due to scratching, a lack of correlation between natural and artificial weathering for the referenced feature. Some of the coating systems they tested, following four years of exposure in natural conditions, were characterised by significantly larger corrosion areas versus the samples subject to weathering for 4–6 months with the use of a cyclic test; for some systems, the corrosion area was larger after the cyclic test versus natural weathering. Looking for a correlation between the corrosion of incised coatings subject to accelerated and natural weathering in marine conditions, Krystel Pélissier et al. [22] demonstrated that in order to observe correlations between the impacts of natural and accelerated weathering, longer (even 5–7 years) exposure in natural conditions is required. Ramdé et al. [23] demonstrated that air humidity and temperature significantly contribute to the colour change in acrylic binder-based waterborne paint samples. Scrinzi et al. [19] investigated the colour change in samples subject to natural and accelerated weathering (cycle: UV test + NSS test), demonstrating quite a good correlation for the parameter. Scientists from Laval University [16] observed that the colour change after tests in weathering chambers featured with fluorescent lamps does not correspond to the actual colour change in natural conditions. Even though UV light is the main contributor to the colour change, other factors, such as exposure angle, additional snowfall, hail and other environmental factors, can considerably affect the parameter. Valverde and Moya presented similar conclusions in their study [24], paying attention to the fact that it is hard to set the parameters of artificial weathering in UV chambers to simulate natural weathering in a specific geographical area. Solar radiation, temperature and air humidity are highly volatile in natural conditions, whereas we can strictly control the parameters in weathering chambers. Juan and Roger [24] also indicated that, in addition to the variability in climate conditions, there are other natural factors, e.g., living organisms, which accelerate coating degradation, including colour change.

Considering the above, planning laboratory tests concurrently simulating many environmental factors seem necessary. Cyclic tests are an example of such a solution, and their execution can help researchers simulate the durability and corrosion resistance of paint systems considering the target environment’s conditions of use. The task poses a challenge due to the diversity and variability in environmental conditions, even in the same country. Further research is indispensable to improve accelerated weathering methods, including but not limited to new groups of corrosion protection coatings and the correlation of accelerated and natural weathering [25]. The literature presents many results of studies demonstrating a correlation between natural and artificial degradation for specific coating parameters, e.g., electrochemical impedance spectroscopy (EIS) and Fourier-transform infrared spectroscopy (FTIR). Still, some results contradict this thesis [7,17,19,22,25,26]. A major problem in obtaining a correlation between natural and artificial weathering is related to limited amounts of data. Even accelerated corrosion tests are time-consuming and costly, so they do not generate many samples, and, consequently, such methods as neural networks cannot be applied [2]. Scrinzi et al. [19] demonstrated that in order to understand coating degradation in natural conditions, it is essential to record environmental parameters—next to exposure time—such as solar radiation, atmospheric precipitation, temperature, humidity and pollution, since the parameters do not act individually but degrade organic coatings in synergy [27].

**Figure 1:**
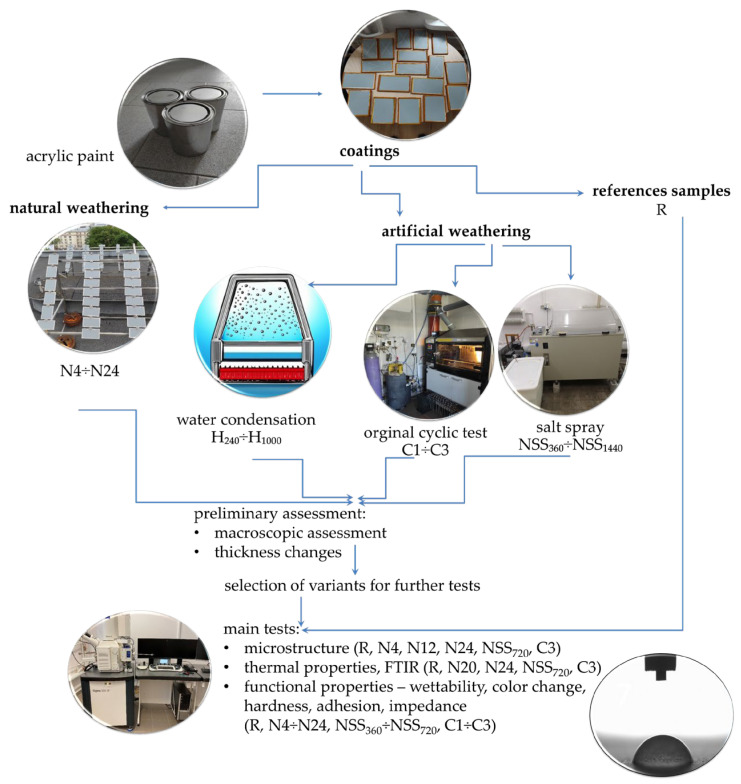
Experimental flowchart with photos.

Methods for testing the resistance of waterborne coatings to environmental conditions have not been determined so far. That is why this study attempts to assess the fitness of standard accelerated weathering methods for corrosion protection in the new category of the coatings mentioned above, which has not been analysed so far. In addition, the unprecedented original cyclic test was developed that simulates the impact of a broad spectrum of environmental factors: UV radiation, condensation humidity, neutral salt spray, negative and elevated temperature, and sulphur dioxide. An analysis was performed of the changes in waterborne acrylic coating characteristics under the influence of artificial weathering carried out with different techniques. The changes observed in accelerated tests were those observed during exposure in natural conditions. Macroscopic and microscopic evaluations were carried out, and thermal properties and spectra were analysed with FTIR. Moreover, the utility properties of the coatings were evaluated for their thickness, colour, adhesion and impedance. A flowchart of works versus the whole experiment is summarised in Figure 1.

## 2. Materials and Methods

### 2.1. Coatings

A coating system consisting of two layers of one-component acrylic waterborne paint, whose main components are presented in Table 1, was the test subject. Table 2 summarises the physical characteristics of the paint. The paint system used in the tests is intended for corrosion protection of steel constructions in traffic structures, e.g., bridges and flyovers.

The paint was applied to S235JR structural steel panels, prepared to Sa 2 ½ grade according to ISO 8501-1 [28]. The application was performed for all samples under the same conditions using the airless spray method. It was executed by an experienced company specialising in structural steel corrosion protection services. The coatings were 200–210 μm thick.

**Figure 2:**
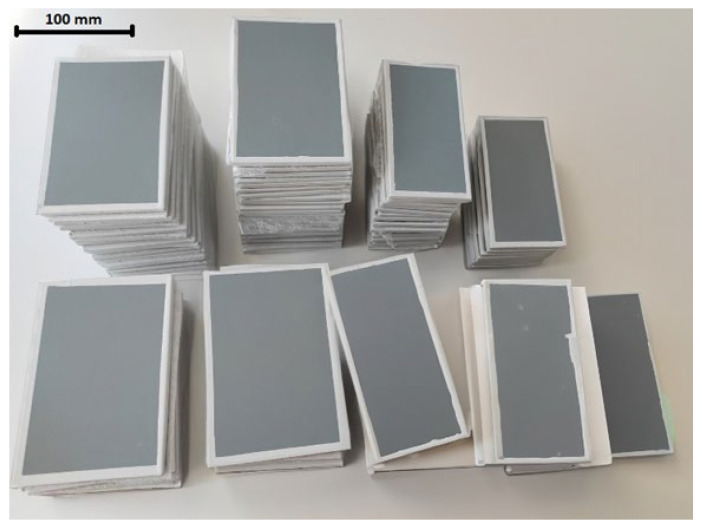
Test samples—steel panels with acrylic paint coating.

One hundred 150 × 100 × 5 mm panels and one hundred 150 × 75 × 5 mm panels were prepared for the tests. The panel edges were protected with an extra coating layer and epoxy resin/paraffin to intensify corrosion protection of sensitive areas. A general view of selected panels is shown in Figure 2. On some panels meant for scratch corrosion evaluation tests, 100 mm long and 1 mm wide X-shaped scribes (incisions) were made. The coating was incised up to the steel substrate depth. The panels were conditioned under laboratory conditions (temp. 23 ± 2 °C, 50 ± 5% RH) for thirty days from paint application.

### 2.2. Exposure to Natural Weathering

**Figure 3:**
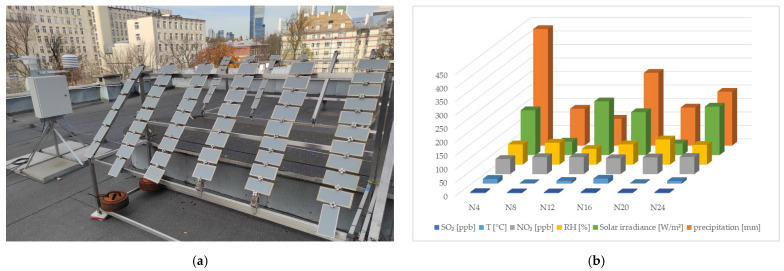
Natural weathering: (**a**) test site with test panel frames and weather station, (**b**) environmental conditions recorded by the weather station. The data cover 24-month exposure and are presented either as a sum (precipitation) or median (other), recorded during 4 months (N4), 8 months (N8) and 24 months (N24).

A half of the panels were subject to natural weathering. This involved exposure on a test site (Figure 3a) in a C3 corrosion-category environment [29]. The test site was situated on the flat roof of the Building Research Institute, on the fourth floor, with geographical coordinates 52°13′ N 21°0′ E. The panels were fixed to frames (display stands) made of stainless steel and wood. The samples were positioned at 60° against the surface. A weather station Meteo LB-490, (LAB-EL, Reguły, Poland) was placed in the direct vicinity of the samples. The weather station was supposed to record environmental conditions, i.e., temperature, air relative humidity, rainfall, solar radiation intensity and NO_2_ and SO_2_ content. The data were recorded in ten-minute intervals. The exposure on the test site and registration of environmental conditions were carried out for twenty-four months, from June 2021 to June 2023. The weather station data are summarised in Figure 3b. All parameters except for precipitation are presented as a median. The median of the solar radiation intensity covered only the period with intensity values > 0. Atmospheric precipitation is presented as a total for the given period. The test series are marked as N4, N8… and N24, where the numerical component stands for natural weathering time in months, i.e., 4, 8… and 24 months, respectively.

At each exposure stage, macroscopic evaluation and thickness measurements were performed, and series were selected for performance testing. The microstructure was analysed after selected stages (N4, N12 and N24). A thermal analysis and FTIR were performed in the final exposure stage (N20 and N24). Non-destructive tests—thickness, colour and contact angle measurements—were carried out after each exposure stage for the same samples. The samples were cleaned with demineralised water and then conditioned for six hours under laboratory conditions. After the tests, the samples were placed back in the display stands. Samples for other tests were removed in groups, cleaned with water, conditioned under laboratory conditions for at least seven days and tested.

### 2.3. Exposure to Artificial Weathering

Accelerated (artificial) weathering was carried out simultaneously. The exposure is standard for solvent-based paints and concerns water condensation impact according to ISO 6270-1 [30] and neutral salt spray atmosphere according to ISO 9227 [31]. Moreover, an original cyclic test was programmed, considering a combination of a broad range of factors used in artificial weathering polymer-based coating products [17,20]. In all cases, the exposure was executed in stages, as summarised in Table 3.

Exposure to condensation (H) was executed in a condensation chamber (Operations Technology Institute, Radom, Poland). It involved continuous exposure to water condensate at 38 ± 2 °C. The samples were exposed at 20° against the substrate level. The conductivity of the mineralised water used amounted to 10 µS/cm.

The NSS test was performed in the CCT 1000-TL-D chamber (VLM GmbH, Bielefeld, Germany). It involved creating a salt spray atmosphere in the chamber’s working space using 5% NaCl. The samples were exposed at 70° against the substrate. The temperature in the chamber during the test amounted to 35 ± 2 °C.

The original cyclic test (C) considered a combination of a broad range of factors used in the artificial weathering of polymer-based coating products, i.e., UV radiation, temperature, humidity and neutral salt spray atmosphere [17,20]. The test was completed with the factor representing environmental pollution, which is rarely used in testing. The pollution was imitated by using a humid atmosphere containing SO_2_, causing significant coating degradation [15,20] and occurring in natural conditions. The original cyclic test was composed of three stages, each consisting of 120 h of exposure to UV irradiation and water condensation according to ISO 16474-3 [32], 168 h of NSS test according to ISO 9227 [31], 24 h of exposure to negative temperature and two cycles of Kesternich test according to ISO 3231 [33]. Table 4 summarises the details of the original cyclic test. The exposure was first performed in the salt chamber mentioned above and then in the QUV spray chamber (Q-LAB, Westlake, OH, USA) at 60° against the substrate. Exposure to negative temperature was carried out in a climate chamber (Weisstechnik GmbH, Reiskirchen-Lindenstruth, Germany) by placing the samples in the chamber vertically. The exposure to a humid atmosphere containing SO_2_ was performed in the CON 300 FL chamber (VLM GmbH, Bielefeld, Germany). The samples were positioned at 70° against the substrate.

Each exposure stage (H, NSS and C) was followed by macroscopic evaluation and thickness measurement. Moreover, some series were selected for performance tests. Considering the steel substrate’s corrosion grade and almost complete degradation of the polymer coating, further testing of the NSS_1440_ series samples was abandoned. At the final stage of exposure in the salt spray atmosphere (NSS_720_) and according to the original cyclic test (C3), the microstructure was analysed and thermal analysis and FTIR performed. Non-destructive tests—thickness, colour and contact angle measurements—were carried out after each exposure stage. The samples were cleaned with demineralised water and conditioned for 6 h under laboratory conditions. The samples for other tests were removed in groups, cleaned with water, conditioned under laboratory conditions for at least seven days and then tested. The course of the works is presented schematically against the whole experiment in Figure 1.

### 2.4. Macroscopic Evaluation

After each stage of natural and artificial weathering, the coating was evaluated macroscopically in daylight, with the naked eye or using a magnifying glass (magnification ×10), if necessary. The quantity and size of blistering, corrosion, flaking, cracking, chalking and corrosion from the scribe were determined. The results are expressed on a scale according to the series of ISO 4628 [34] standards, sheets from 2 to 6 and 8. In reference to blistering, cracking and chalking, a homogenous system of describing the changes as XS(Y) was used, where X stands for quantity, while Y represents the size of the changes. In both cases, a numerical scale from 0 to 5 was applied. Referring to the quantity, 0 means no changes; 1—very few changes; 2—a few changes; 3—a moderate number of changes; 4—a considerable number of changes; and 5—a dense pattern of damage. Referring to the size, 0 means changes not visible at magnification ×10; 1—visible at magnification ×10; 2—barely noticeable with the naked eye; 3—up to 0.5 mm; 4—from 0.5 mm to 5 mm; and 5—larger than 5 mm.

### 2.5. Thickness

The coating thickness was measured after each stage of weathering exposure, under both natural and artificial conditions. The Phascope PMP10 Duplex (Helmut Fischer, Sindelfingen, Germany) meter was used with the magnetic induction method. Six measurements in uniformly distributed areas, at least 10 mm from the edge, were performed for each sample.

### 2.6. Microstructure Analysis

The microstructure of the coating surface was investigated using a Sigma 500 VP scanning electron microscope with a cold-field emission (Carl Zeiss Microscopy GmbH, Köln, Germany). The microstructure surface of the coatings was examined at an accelerating voltage of 5 KeV excitation electron beam, using an SE detector, on samples sprayed with a coat of gold. The observations were carried out at a magnification of x5000. The microstructure of the coating surface was observed in the initial state (R) after exposure in natural conditions for four months (N4), twelve months (N12) and twenty-four months (N24). In addition, SEM observations were carried out on samples after artificial weathering using salt spray for 720 h (NSS_720_) and after subjecting to the custom test covering three cycles with a total exposure of 1080 h (C3).

### 2.7. Thermal Properties

Thermal properties were tested with thermogravimetry (TGA) and differential scanning calorimetry (DSC). TGA measurements were performed with the Q5000IR apparatus (TA Instruments, New Castle, DE, USA). The samples were placed on platinum pans and heated up to 750 °C, at 10 °C/min heating rate and nitrogen flow rate amounting to 25 mL/min. DSC measurements used Q2000 apparatus (TA Instruments, New Castle, DE, USA). The samples were crimped in Tzero aluminium pans and tested according to a heat/cool/heat procedure in a temperature range between −50 °C and 180 °C, with a heating/cooling rate of 10 °C/min and nitrogen flow rate of 50 mL/min. The TGA and DSC results were processed and analysed with Universal Analysis software (version 4.5A, TA Instruments, New Castle, DE, USA). The samples used for the tests included non-weathered coatings (R), coatings weathered as a result of natural exposure for twenty and twenty-four months (N_20_ and N_24_, respectively), artificially weathered under neutral salt spray for 720 h (NSS_720_) and according to original accelerated weathering cyclic test—three cycles and total exposure time of 1080 h (C3).

### 2.8. Fourier-Transform Infrared Spectroscopy

The FTIR measurements were made with a Nicolet 8700 spectroscope (ThermoScientific, Madison, WI, USA) within an attenuated total reflectance (ATR) mode and diamond crystal. The measurements for all coatings were performed in three different zones. Sixty-four scans with a resolution of 4 cm^−1^ in the 400–4000 cm^−1^ range were collected and averaged. The spectra were recorded, processed and analysed with dedicated Omnic software (version 8.2.0.387, ThermoScientific, Madison, WI, USA). The samples used for the tests included non-weathered coatings (R), coatings weathered as a result of natural exposure for twenty and twenty-four months (N_20_ and N_24_, respectively), artificially weathered under neutral salt spray for 720 h (NSS_720_) and according to original accelerated weathering cyclic test—three cycles and total exposure time of 1080 h (C3).

### 2.9. Performance

After each stage of weathering exposure, both under natural and artificial conditions, parameters, such as adhesion, hardness, contact angle, colour change and impedance, were verified.

An adhesion test was performed with a DY-206 pull-off tester (Proceq, Schwerzenbach, Switzerland) according to ISO 4624 met. B [35]. Six measurements were performed for each series using 20 mm diameter dollies at a pull-off rate of 0.5 MPa/s.

Hardness was tested using a Buchholtz (TQC Sheen, Capelle aan den Ijssel, The Netherlands) hardness meter. Fifteen measurements were made for each series according to ISO 2815 [36].

The contact angle was measured with a Fibro PGX+ goniometer (TQC Sheen, Capelle aan den Ijssel, The Netherlands) by performing six measurements for each series in random areas. A 4 µL demineralised water droplet was used for the test.

The colour change was measured with SP62 spectrophotometer (X-Rite, Grand Rapids, MI, USA). D65 light was used as the light source, 10° calorimetric observer and d8° measurement geometry. Three measurements were performed for each sample in order to record the change in the a* b* colour parameters and L* psychometric brightness. The total colour change was determined as ΔE*_ab_ according to Equation (1).

(1) $${{\Delta E}^{*}}_{\mathrm{ab}}=\sqrt{{\left(\Delta L\right)}^{2}+{\left(\Delta a\right)}^{2}+{\left(\Delta b\right)}^{2}}$$

where: ΔE*_ab_—total colour change; ΔL—difference between L* before and after weathering; Δa—difference between a* before and after weathering; Δb—difference between b* before and after weathering.

Electrochemical impedance spectroscopy (EIS) was carried out with the ATLAS 0441 (ATLAS-SOLLICH, Rębiechowo, Poland) impedance analyser in a standard system with three electrodes. The working electrode was the steel sample with an applied coating in a measurement cell with a 12.57 cm^2^ area. The reference electrode was made of Ag/AgCl, while a platinum wire with a mesh was the counter electrode. The measurement cells were filled with 3% NaCl solution in demineralised water. Thirty minutes after their filling with electrolyte, the coatings’ resistance parameters were recorded. The frequency range in the test was between 10^5^ and 0.1 Hz, and the amplitude value amounted to 100 mV.

All performance tests were carried out on the coating under laboratory conditions (23 ± 2 °C, 50 ± 5%RH).

## 3. Results and Discussion

### 3.1. Macroscopic Evaluation

The results of the macroscopic evaluation of coatings at different stages of both natural and artificial weathering are summarised in Table 5. For no test series, at no observation stage, degradation changes in the form of the coating flaking, cracking or chalking were observed, even at magnification ×10, corresponding to grade 0(S0).

**Figure 4:**
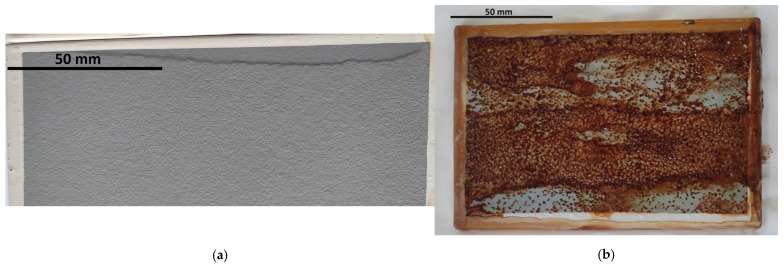
General view of the artificially weathered sample: (**a**) coating swelling after 1000 h of water condensation exposure, (**b**) corrosion after 1440 h exposure to neutral salt spray atmosphere (NSS_1440_).

**Figure 5:**
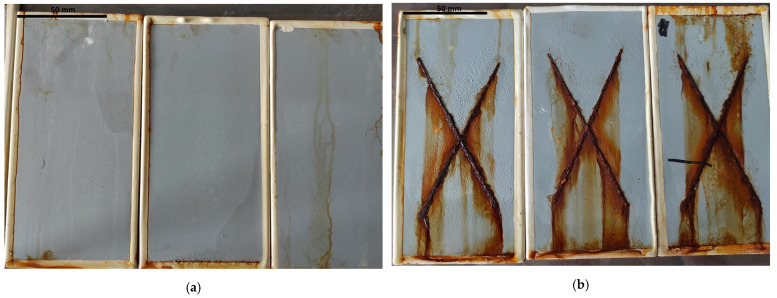
General view of the artificially weathered samples after 3 cycles of original test exposure: (**a**) coating without scribes, (**b**) coating with 1 mm scribes.

Blistering was the dominant type of change. It shall be highlighted that they were not observed at any exposure stage in natural conditions. The N4–N24 series samples revealed no traces of blistering, even at magnification ×10, which corresponded to grade 0(S0). Blistering was visible during artificial weathering but occurred at different stages of selected exposures and with varied intensity. Blistering was not observed at the initial stage of water condensation exposure. A minimal number of changes, noticeable only at magnification ×10—grade 1S(1)—was observed only after 1000 h of exposure (H_1000_). Nevertheless, it shall be emphasised that coating swelling was observed right from the first exposure stage (as presented in Figure 4a and discussed in detail in Section 3.2), making blistering evaluation difficult. The exposure to neutral salt spray caused coating blistering at the very first stage of exposure, whereby for the NSS_360_ series with no scribes, minor blistering was reported, visible only at magnification ×10—2S(1). In the case of incised samples, the damage pattern was dense but barely noticeable with the naked eye—5S(2). The longer the weathering time was, both for samples with and without a scribe, the blistering intensified, respectively, up to grade 3(S2), moderate quantity of blistering barely noticeable with the naked eye, and 5(S3), dense blistering pattern up to 0.5 mm in size. The coating behaved completely differently during exposure in the original cyclic test. Non-incised samples had no traces of blistering throughout the entire exposure period, similar to the C1 series with scribes. A significant quantity of blistering was reported for the incised samples after C2 and C3, barely noticeable with the naked eye, 4(S2). The samples with and without scribes are presented in Figure 5a,b.

Rusting was also observed. The substrate corrosion area for naturally weathered samples (N4–N24 series) and after water condensation exposure (H_240_–H_1000_) equalled 0, Ri0 grade. When observing the samples subject to neutral salt spray, substrate corrosion was reported, which enlarged with longer exposure time. The corrosion area for the NSS_360_ series samples was 1%, Ri3 grade; for the NSS_720_ samples, it was 8%, Ri4, and 40–50%; for the NSS_1440_ samples, Ri5 grade (Figure 4b). For the samples subjected to the original cyclic test, substrate corrosion was observed only for the C3 series—Ri1 grade.

The width of corrosion around the scribe for the N4–N24 samples was 0 mm. Corrosion around the scribe was not evaluated for H_240_–H_1000_ water condensation exposure. The corrosion around the scribe for the NSS_360_ and NSS_720_ samples was 0.5 mm, while for the NSS_1440_ samples, it amounted to 2 mm. After the cyclic test on the C1 series samples, no corrosion around the scribe was observed, while the corrosion of the C2 and C3 series samples was 1 mm wide.

The analysis of the coating macroscopic observations conducted at different weather stages, both natural and artificial, indicates that exposure to neutral salt spray was the most destructive for the acrylic coating. Blistering and rusting were observed already at the initial exposure stage (NSS240), and they intensified significantly during successive exposure stages. The degree of rusting observed in the final stage (NSS_1440_) prevented further coating examination. It should be emphasised that such intensive changes, noticeable from the early exposure stage, were not observed for any other weathering. Significant similarity to the coating’s behaviour in natural conditions (N4–N24) was, however, observed in the behaviour of the non-incised coating during the original cyclic test (C1–C3) and weathering with the use of condensation water (H_240_–H_1000_). In all cases, no degradation changes noticeable with the naked eye were observed. The scribe of the coating increased its susceptibility to degradation. A higher degree of changes was observed both for the series weathered with a neutral salt spray atmosphere (NSS_360_) and according to the original cyclic test (C2, C3). It is most likely related to the electrolyte penetration running from the scribe deep into the sample, which promotes osmosis through the coating [37].

### 3.2. Thickness

**Figure 6:**
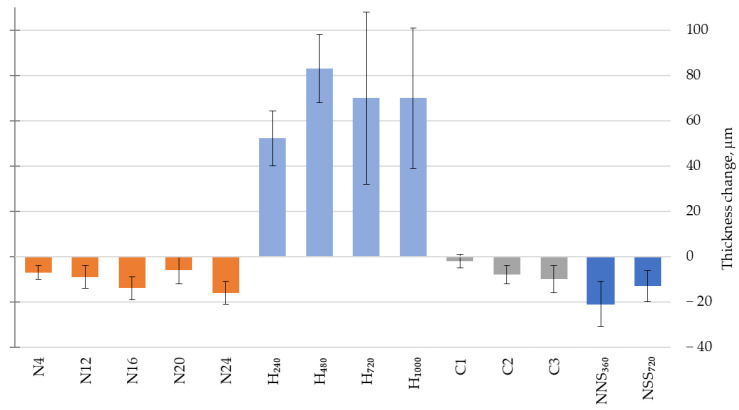
Change in the coating thickness after ∎ natural weathering for a period between 0 and 24 months, including intermediate evaluation after 4, 12, 16, 20 and 24 months (N4–N24); ∎ artificial weathering by water condensation for a period between 0 and 1000 h, including intermediate evaluation after 240 h, 480 h and 720 h (H_240_–H_1000_); ∎ after artificial weathering according to the original cyclic test between 1 (C1) and 3 (C3) cycles; ∎ artificial weathering in a neutral salt mist for a period between 0 and 720 h, including intermediate evaluation after 360 h (NSS_360_–NSS_720_); the error bars represent standard deviation.

An analysis of the coating thickness measurement results reveals that both natural and artificial weathering cause changes that aggravate with the increasing exposure duration (Figure 6). For the coating exposed in natural conditions (N4–N24), exposed to a neutral salt spray atmosphere (NSSs_360_–NSS_720_) and subjected to the original cyclic test (C1–C2), reduced thickness was observed, which matches previous study results [11,26,38]. The reduction did not exceed 10% of the original value, which amounted to ca. 200 μm. The decrease in the polymer coating thickness may be related to erosion caused by precipitation and wind-transferred mechanical pollution [39,40], additionally accelerated by the impact of UV radiation [41].

The coating thickness being higher for the N20 series than for N24 is most likely related to different weather conditions during the measurement. The highest relative humidity median amounting to 93% at a relatively low temperature of ca. 3 °C was observed during the N20 series measurements (Figure 3b). A significant thickness decrease was reported for the N24 series samples, corresponding to the trend observed throughout the exposure period of 0–24 months. It should be highlighted that during the N24 measurements, the median of air relative humidity was ca. 70% RH, and the air temperature was ca. 10 °C (Figure 3b). Considering that waterborne paint systems tend to absorb water [13,14], which might cause their blistering [37], a higher thickness after exposure to humid conditions seems justified. An acrylic coating’s susceptibility to swelling due to a long-term water impact was demonstrated by its thickness, higher by 50–80 μm after artificial weathering using condensation water (H_240_–H_1000_). A thickness increase of 25–40% against the initial state was observed in this test but not for any other exposures. Considering the significant difference against other exposures, especially in comparison with natural conditions, and simultaneously taking into account some technical constraints in performing tests on such a swollen coating, a decision was made to abandon further testing on the H_240_–H_1000_ series. Based on the observations above, a weathering test involving long-term exposure to condensation humidity required a separate, in-depth analysis of its fitness for evaluating acrylic coatings. The impact of time and samples’ seasoning conditions between the end of exposure and making the measurement is among the factors to be analysed. It seems that under adequate conditions, water evaporates from the coating, and the observed thickness increase after condensation tests can be temporary [13].

### 3.3. Microstructure Analysis

Waterborne coatings degrade during use and exposure to a corrosive environment, which is accompanied by changes in microstructure. The impact of factors characteristic of the conditions of use of corrosion protection coatings for steel structures includes mechanical loads, climate conditions (UV radiation, temperature fluctuations, water), the corrosive impact of the atmosphere and microorganisms contribute to the degradation of polymer coatings due to polymer chain breaking [8,34]. That is why this study evaluates the surface quality and verifies surface damage by analysing the coating’s morphology in the initial state (R), after natural weathering (N4, N12, N24), weathered artificially in the salt spray atmosphere (NSS_720_) and according to the original cyclic test (C3).

**Figure 7:**
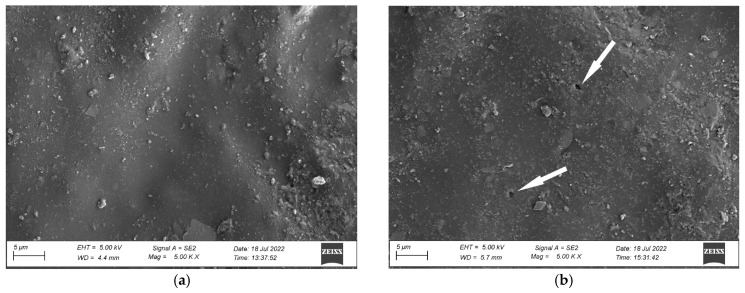
Microstructure of the coating’s surface: (**a**) initial state (R), (**b**) after natural weathering for 4 months (N4); pitting is marked by an arrow; magnification ×5000.

**Figure 8:**
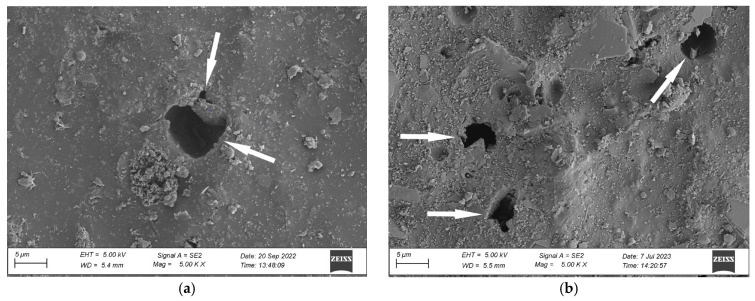
Microstructure of the coating’s surface after natural weathering for (**a**) 12 months (N12), (**b**) 24 months (N24); pitting is marked by an arrow; magnification ×5000.

An analysis of the coating’s surface microstructure in the initial state revealed a uniform and continuous coating (Figure 7a). The images show only minor contamination of the coating and particles of mineral fillers. Exposing the coating to natural environmental conditions for four months (N4) resulted in slight changes in the microstructure. The SEM revealed small pits with sizes around 1 µm (Figure 7b). The surface morphology analysis, carried out after twelve months of exposure (N12), demonstrated pits with sizes ranging from 5 µm to 10 µm and smaller pits in a size range between 1 µm and 2 µm (Figure 8a). The presence of these pits clearly shows polymer degradation [42,43]. Moreover, significant contamination of the material surface can be observed in the images. The change in the coating’s microstructure under an environmental impact can harm the performance of the analysed materials [40,43].

Further exposure to natural conditions for twenty-four months (N24) did not intensify the coating’s surface degradation. The images show pits with sizes ranging from 1 µm to 6 µm (Figure 8b), i.e., smaller ones than after twelve months of exposure. Smaller sizes of the coating defects can result from the difference in the atmospheric conditions during sampling. For the samples weathered on the test site, the microstructure change can be attributed to the impact of UV radiation, erosion due to rain and hail, and wind-transferred mechanical pollution [40]. Microscopic observations confirm the results of measurements of utility properties presented further in this study and show a correlation between surface degradation and material properties. The samples subjected to natural weathering reveal degradation changes such as contamination anchored in the material and the presence of voids and larger pits.

**Figure 9:**
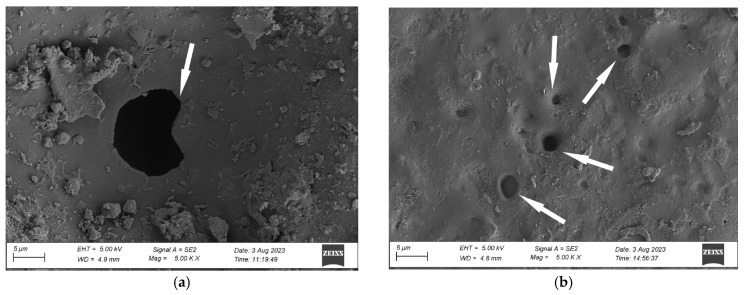
Microstructure of the coating’s surface after (**a**) exposure to neutral salt spray (NSS_720_), (**b**) original cyclic test (C3); pitting is marked by an arrow; magnification ×5000.

In the next stage of work, the coating’s microstructure after exposure to natural conditions was compared with the microstructure after artificial weathering, including exposure to neutral salt spray (NSS_720_) and according to the original cyclic test (C3). The results of microscopic observations reveal a significantly higher degradation rate of the coating after exposure to salt spray. The images show voids with sizes ranging from 15 µm to 20 µm (Figure 9a). The NSS_720_ series coating degradation was more significant than after the cyclic test, where the pit sizes ranged from 1 µm to 5 µm (Figure 9b).

The coating’s microstructure changes after artificial weathering according to the original cyclic test C3 (Figure 9b) were similar to those reported for the N_12_ series samples naturally weathered for twelve months (Figure 8a) and N_24_ series samples weathered for twenty-four months (Figure 8b).

### 3.4. Thermal Characteristics

**Figure 10:**
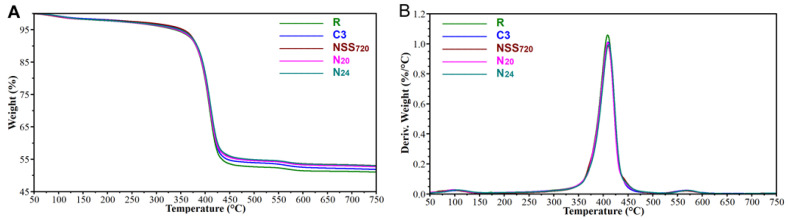
TGA (**A**) and DTGA (**B**) curves recorded for the tested coatings.

The TGA measurements were made to evaluate the potential changes both in the examined coatings’ thermal stability and the inorganic filler content. Macroscopic evaluations revealed that changes may have occurred in the materials, which was suggested by the presence of pores on their surface. The results of the analyses are summarised in Figure 10 and Table 6.

Three stages of mass loss were observed for all coatings. The first stage, progressing most intensively for temperatures of ca. 100 °C, is related to the dehydration of the coatings and the potential evaporation of additives with low thermal stability, e.g., 2-butoxyethanol (cf. Table 1). The mass loss corresponding to the process was minor, within a 1.6–1.9% range. Then, a further slow mass loss was observed, related to the formation of anhydrides from carboxylic acids in the polymer or the loss of hydrogen atoms from the main chain [44]. The second significant decomposition stage occurs within a temperature range of ca. 350–450 °C and is related to the primary degradation of the acrylic matrix. The third decomposition stage, characterised by mass losses of 1.4–1.7%, occurring within a temperature range of ca. 525–600 °C, is the result of matrix residue carbonisation [44]. The total mass loss related to polymer degradation (marked as m_2_ in the Table), i.e., covering anhydrite formation, hydrogen atom loss from the primary chain and main pyrolysis, was most significant for non-weathered samples (R) and alleviated for naturally weathered (N_20_ and N_24_) and artificially weathered samples, both in the salt spray atmosphere (NSS_720_) and according to the original cyclic test (C3). Consequently, the share of inorganic fillers changed, which was identified after pyrolysis carried out up to 750 °C. The observed reduction in the polymer fraction content suggests that the erosion rate of the polymer coating matrix is much higher than that of the inorganic filler.

It can be noticed that as a result of long-lasting natural and artificial weathering, the thermal stability of the analysed coatings did not change significantly from an application point of view. Some discrepancies in the identified T_d5%_ values can be attributed to different water contents in the coatings.

**Figure 11:**
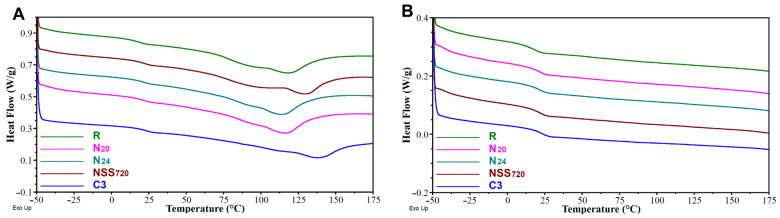
DSC curves recorded for 1st (**A**) and 2nd (**B**) heating cycles of non-weathered and artificially weathered coatings.

The results of the DSC analysis are summarised in Table 7 and Figure 11. For all analysed coating states, in the first heating cycle, a transition from a vitreous to a visco-elastic state (vitrification) was observed, accompanied by a broad endothermic peak of a more or less noticeable but always bimodal nature. Since it occurs only in the first cycle, it is most likely caused by water evaporation and also by evaporation or melting of the additives (e.g., 2-butoxyethanol). The vitrification temperature was observed to increase slightly due to coating weathering. This suggests that a coating-softening ingredient is washed out. The identified temperatures were slightly lower in the first heating cycle, which can be explained by the presence of water and/or other additives acting like plasticisers. The effect observed for the non-weathered sample was the opposite, i.e., T_g_ was slightly lower in the second cycle. From an application point of view, the results do not reveal any significant differences between the materials.

### 3.5. FTIR

**Figure 12:**
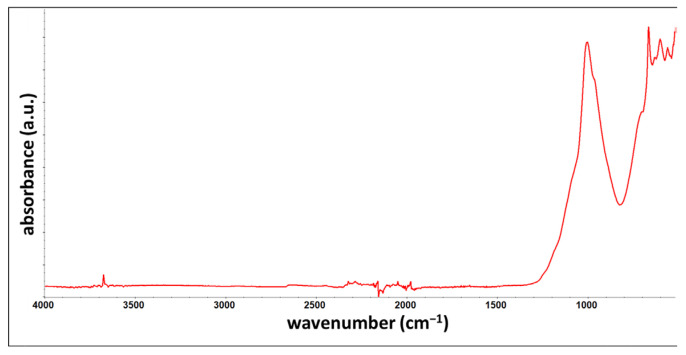
Spectrum recorded for residues of pyrolysed non-weathered coating.

The coatings were also tested using Fourier-transform infrared spectroscopy (FTIR). The spectra of inorganic pyrolysis residues were additionally recorded (Figure 12). Titanium dioxide should be the primary inorganic filler (Table 1). Nonetheless, the recorded spectrum is not typical of the referenced compound [45,46] talc spectrum [47,48]. The most intensive band with a peak at 1004 cm^−1^, similar to the peak at 666 cm^−1^, originates from the Si-O stretching vibrations, whereas the band at 3675 cm^−1^ originates from stretching of O-H surrounded by the MgMgMg triplet [49,50].

**Figure 13:**
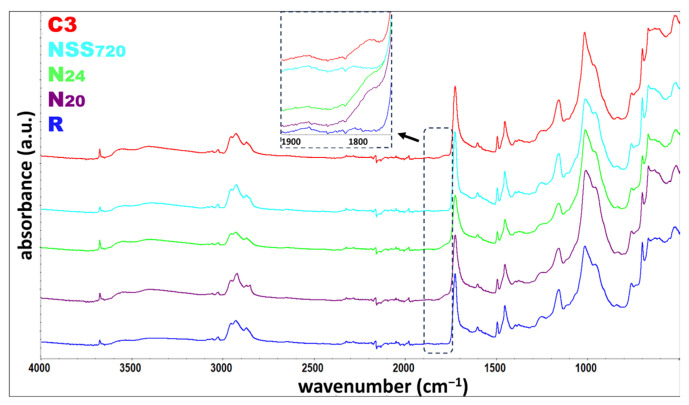
ATR-FTIR spectra recorded for non-weathered and artificially weathered coatings.

The spectra recorded for the non-weathered coating and coatings weathered in natural conditions (N20 and N24) and artificially weathered, in the salt spray atmosphere (NSS720) and according to the original cyclic test (C3) (Figure 13), did not reveal significant differences between samples of different states. The positions of the main characteristic bands (Table 8) did not change. Nonetheless, a thorough analysis showed that as a result of coating weathering, the intensity ratio of bands characteristic for the filler (~1011 cm^−1^ and 3675 cm^−1^) to the intensity of the band characteristic for the polyacrylic matrix (i.e., 1725 cm^−1^, 1452 cm^−1^, 1158 cm^−1^, and 698 cm^−1^) rises. This is testimony for the reduced share of the polymer phase in the coating, which complies with the TGA results. Most importantly, for the naturally weathered coatings and those exposed to artificial weathering according to the original cyclic test (C3), broadening was observed at the peak base from the C=O stretching vibrations, as shown in Figure 13. The effect is caused by the formation of photodegradation products under UV radiation [51]. The effect was not observed for the coating weathered in a salt spray environment (NSS_720_).

Bearing in mind the material characteristics described above, the most relevant information on the possibility of representing natural weathering on the test site in laboratory tests is obtained from the FTIR analysis. It unequivocally reveals that applying a cyclic process triggers material changes identical to those in samples exposed to natural external conditions.

### 3.6. Performance

**Figure 14:**
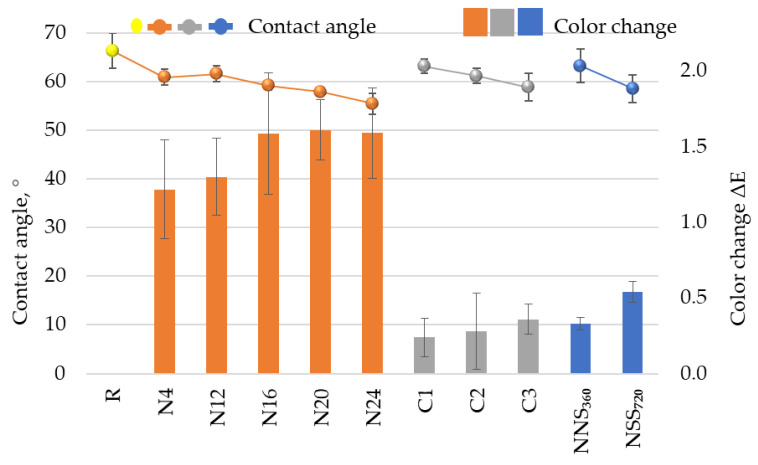
Change in the colour and contact angle ● reference samples, ∎ natural weathering for a period between 0 and 24 months, including intermediate evaluation after 4, 12, 16, 20 and 24 months (N4–N24), ∎ artificial weathering according to the original cyclic test—between 1 (C1) and 3 (C3) cycles, ∎ artificial weathering in a neutral salt mist for a period between 0 and 720 h, including intermediate evaluation after 360 h (NSS_360_–NSS_720_); the error bars represent standard deviation.

Natural weathering resulted in a contact angle change (Figure 14). Data analysis for the N4–N24 sample series shows a declining trend. The contact angle for N24 samples dropped by 11° compared to the reference sample R. A similar trend was observed for the C1–C3 series samples. The NSS_720_ samples were characterised by contact angles similar to those of N_24_ and C3. The decrease in the contact angle value in the function of time suggests an increase in the coating’s hydrophilic nature due to weathering exposure. The coating’s increasing wettability means its higher susceptibility to absorb moisture [3], consequently reducing its corrosion protection capability due to a larger contact area with corrosive media. The contact angle value slightly deviating from the general trend for the N12 series could be related to the atmospheric conditions during the measurement. The air relative humidity median reported for the measurement period was the lowest for the entire exposure duration, i.e., ~58% RH, whereas the solar radiation intensity was the highest ~200 W/m^2^; moreover, the total precipitation in the referenced period was the lowest—~100 mm (Figure 3b). Analysing the contact angle values and changes in the coating thickness against the environmental conditions recorded by the weather station, one can observe the relationship between the measurement results and temporary conditions on the test site. The observations confirm the changing nature described in the literature, hampering test programming with accelerated methods [27].

The coating colour change observed in this study was adversely correlated with the changes in the contact angle (Figure 14). For the N4–N24 series samples, the total colour change ΔE*_ab_ amounted to 1.2–1.5, which is considered a very low value. A change of fewer than five units is unnoticeable to the human eye [19,26]. Under artificial conditions, a gradual colour change was observed with longer exposure times for the C1–C3 and NSS_360_–NSS_720_ series samples, but it was over three-times lower than the change for the N4–N24 series samples. Considering the above, it can be stated that the set exposure under artificial conditions, despite taking into account the factors primarily responsible for the colour change in waterborne paint samples, i.e., UV radiation, elevated temperature and humidity [37,52], did not exert an impact similar to that during natural weathering, even in comparison with the first four months of exposure. Exposure to neutral salt spray, for which a correlation with natural weathering was reported [19] for the colour change, did not bring the expected convergence. The authors of other studies [16] indicated that the colour change after tests in weathering chambers featured with fluorescent lamps may not correspond to the actual colour change in natural conditions. Even though UV radiation is essential to colour changes, a different exposure angle, snowfall and other environmental factors can significantly contribute to the parameter change. The colour change measurement results collected during this study suggest a need to consider future modifications to the cyclic weathering test, e.g., by more prolonged exposure to UV radiation and humidity. Due to high fluctuations in solar radiation intensity in natural conditions (Figure 3b), accelerated weathering involving UV radiation is hard to simulate in a laboratory. The authors of [20] reached similar conclusions, pointing out that accelerated weathering parameters are hard to select in UV chambers to simulate natural weathering in a specific geographical area. In natural conditions, solar radiation, temperature and air humidity are highly variable, whereas the parameters can be strictly controlled in weathering chambers. It was also highlighted that in addition to variable climate conditions, other factors that accelerate colour degradation occur naturally, e.g., living organisms.

**Figure 15:**
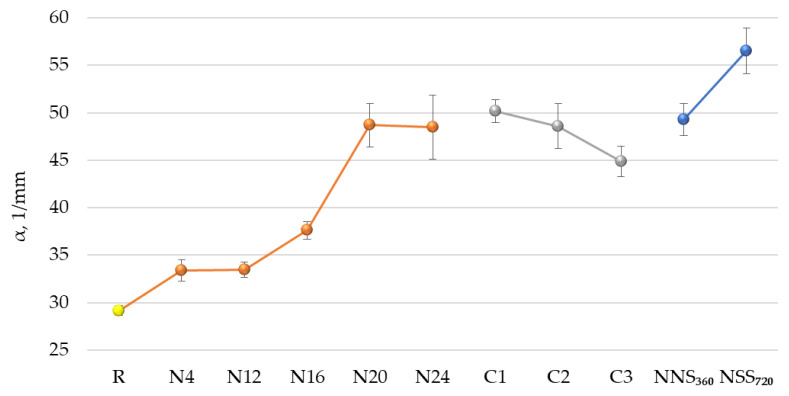
Coating hardness α ● reference samples, ∎ natural weathering for a period between 0 and 24 months, including intermediate evaluation after 4, 12, 16, 20 and 24 months (N4–N24), ∎ artificial weathering according to the original cyclic test—between 1 (C1) and 3 (C3) cycles, ∎ artificial weathering in a neutral salt mist for a period between 0 and 720 h, including intermediate evaluation after 360 h (NSS_360_–NSS_720_); the error bars represent standard deviation.

An analysis of hardness test results (Figure 15), expressing the coating’s susceptibility to denting, reveals that exposure in natural conditions increases the hardness, which is illustrated by the results obtained for the N4–N24 series. For the C1–C3 series samples, a slight decrease in hardness was observed with every cycle. Still, it should be highlighted that the results obtained in the original test were similar to those reported for the N20 and N24 series. The coating hardness for the NSS_360_ and NSS_720_ series samples increased twice against the reference sample, reaching values considerably higher than those for N20–N24.

The hardness increases and decreases observed for the N4–24 sample series are most likely related to momentary atmospheric conditions on the test site when the samples were collected. A noticeable hardness drop observed during the cyclic test was caused by the impact of a low concentration of sulphurous acid formed in a humid atmosphere during the SO_2_ test (H_2_O + SO_2_ → H_2_SO_3_). The sulphurous acid concentration in the accelerated test was much higher than in natural conditions (Figure 3b) and contributed to accelerated coating oxidation, decreasing the coating hardness [53]. The above suggests a need to consider future modifications in the original cyclic test by reducing the SO_2_ concentration.

**Figure 16:**
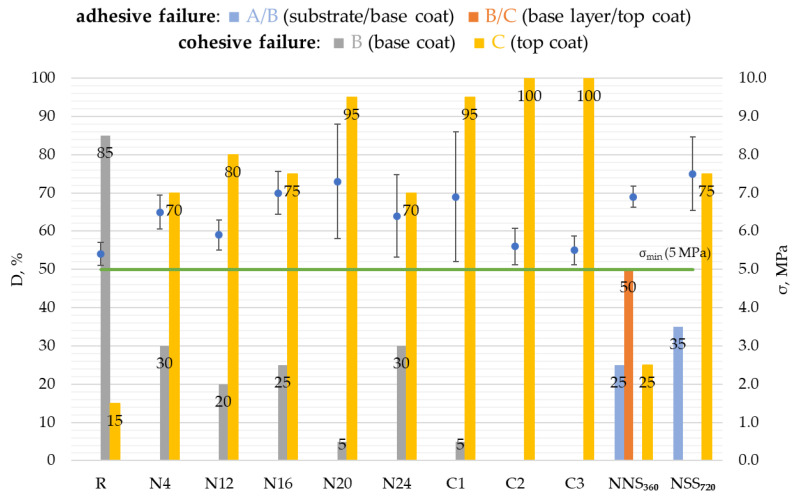
Results of adhesion tests expressed by the bond stress level (σ) and nature of failure (D) obtained for samples weathered naturally for a period between 0 and 24 months, including intermediate evaluation after 4, 12, 16, 20 and 24 months (N4–N24), artificially weathered according to the original cyclic test for 1 (C1) to 3 (C3) cycles, artificially weathered in neutral salt mist for a period between 0 and 720 h, including intermediate evaluation after 360 h (NSS_360_–NSS_720_); the error bars represent standard deviation.

The adhesion of coatings after natural weathering ranged from 5.9 MPa to 7.3 MPa (Figure 16). During exposure in natural conditions (N4–N24), no decrease in the bond stress was reported compared to the initial state (R) values. After artificial weathering, the bond stress for the NSS_360_ and NSS_720_ series samples ranged from 5.6 MPa to 6.9 MPa, while after natural weathering for the C1–C3 series, it was between 6.9 MPa and 7.5 MPa, i.e., at a level not lower than in the initial state.

The nature of the samples’ failure in the adhesion test is particularly interesting. In the initial state (R), after exposure to natural conditions (N4–N24) and after accelerated weathering according to the original cycle (C1–C3), cohesive failure was observed, though it occurred in different layers (Figure 16). For the initial state, failure in the base coat (B) dominated (85%). After N4–N24 weathering, top-coat (C) failure prevailed (70–95%) but with a noticeable share of the base coat (B). After C1–C3, the failure share shifted significantly towards the top coat (C), reaching values over 90%. The breaking nature of the NSS_360_–NSS_720_ samples differed significantly. Cohesive–adhesive failure was observed. Adhesive failure dominated for the NSS_360_ samples, 25% between the base coat and the steel substrate (A/B) and 50% between the base coat and top coat (B/C). The remaining 25% was cohesive failure within the top coat (C). After extending the exposure time for the NSS_720_ samples, a significant (35%) share was observed of the base coat separation from the substrate with steel corrosion exposure and a high (75%) share of the top-coat (C) failure.

By analysing the adhesion test results, one can observe the impact of environmental conditions on the periodic changes of this property. The lowest air humidity medians and the highest solar radiation intensity medians were recorded for the N12 and N24 periods (Figure 3b), which could contribute to tiny drops in the coating’s adhesion to the substrate. The adhesion values obtained for the samples’ exposure to weathering factors under artificial conditions (C1–C3, NSS_360_–NSS_720_) differed only slightly from the reference samples. It should be emphasised that the stress values for all tested variants exceeded the 5 MPa threshold considered satisfactory for steel structure corrosion protection coatings [4]. Moreover, analysing the nature of the coating failure after the adhesion test, it can be noticed that it was similar for N4–N24 natural weathering and C1–C2 accelerated weathering in the cyclic test. Cohesive failure (breaking) of the samples occurred in the base coat or top coat. Adhesive failure of the coating was, in turn, observed for the samples after a neutral salt spray test (NSS_360_–NSS_720_).

**Figure 17:**
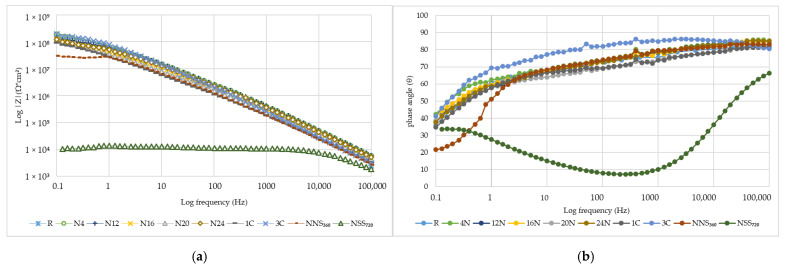
Coating impedance parameters for naturally weathered samples for a period between 0 and 24 months, including intermediate evaluation after 4, 12, 16, 20 and 24 months (N4–N24), artificially weathered according to the original cyclic test—from 1 (C1) to 3 (C3) cycles, artificial weathering in a neutral salt mist for a period between 0 and 720 h, including intermediate evaluation after 360 h (NSS_360_–NSS_720_); Bode plot; impedance module log|Z| (**a**), phase angle (**b**).

The coating’s impedance parameter results are shown in Figure 17a,b as Bode plots [54]. The impedance modules and phase angle suggest that the impedance of the naturally weathered samples (N4–N24) remained at a similarly high level over 1 × 10^8^ Ω cm^2^, and the initial value of the phase angle was 80°. The coating’s resistance to ion penetration is reached when the impedance module value at low frequencies exceeds 10^8^ Ω cm^2^ [7]. Only a minor impedance drop was observed at a low frequency (|Z|_0.01Hz_) for the N4–N24 samples as weathering time progressed. In laboratory tests, the impedance module and phase angle values for the C1–C3 series samples were highly similar to those obtained for the N4–N24 series samples. The samples exposed to neutral salt spray were characterised by lower impedance values. For the NSS_360_ samples, at frequencies < 1 Hz, the curve flattening was observed at 10^7^ Ω cm^2,^ as well as a significant decrease in the phase angle value up to 20°. The measurement results of the impedance module for the NSS_720_ samples revealed a loss in the coating’s barrier function, even at high frequencies, which was demonstrated by the impedance module values of 1 × 10^4^ Ω cm^2^ and a violent drop in the phase angle.

A slight decrease in the impedance module at 0.01 Hz for the N4–N24 naturally weathered samples is a testament to slow degradation changes occurring in the coating’s barrier mechanism, e.g., as a result of electrolyte penetration and enlargement of the pores [55], which was observed in microstructure tests (Figure 8a,b). The impedance values |Z|_0.01Hz_ correspond to the resistance generated by the coating due to electrolyte flow to the substrate; the higher the values, the better the coating’s barrier effect is. Consequently, when the electrolyte penetrates towards the substrate, the value decreases [23]. The drop in the impedance module and phase angle for the NSS_360_ and NSS_720_ samples confirm macroscopic observations, where the first traces of the steel substrate corrosion occurred due to the presence of aggressive ions that accelerated corrosion; the coating’s microstructure (Figure 8b) revealed pore sizes higher than for other series. Analysing the test results, it can be concluded that similarities occur in |Z|_0.01Hz_ for the N4–N24 naturally weathered samples and after the cyclic test (C1–C3). Moreover, the EIS test results reveal that the NSS causes too severe a coating degradation. Similar conclusions were drawn by the researchers, who discovered that the salt spray test caused more considerable coating degradation than natural weathering [19]. A similarity was observed only for exposure in marine environments [17,56].

## 4. Conclusions

Analysis of the study results leads to the conclusion that the degradation of acrylic paint coatings after artificial weathering, performed using methods established for solvent-based coats, differs significantly from that observed in natural conditions. Exposing waterborne coatings to the long-lasting impact of salt spray and condensation humidity caused blistering, rusting and swelling, respectively, much more significant than those observed during exposure on the test site. The above challenges the fitness of the methods for evaluating waterborne paint coatings.

An alternative weathering method was developed in this study. An original cyclic test simulated the impact of a broad spectrum of environmental factors, including UV radiation, condensation humidity, neutral salt spray, negative and elevated temperature, and sulphur dioxide. For waterborne coatings, the test triggered changes similar to those observed in the natural environment. In none of the cases was degradation in the form of flaking, cracking, blistering or chalking confirmed through visual evaluation. Minor rusting was observed only around the scribe. Surface morphology analysis, performed with scanning electron microscopy, revealed the formation of up to 5 μm pits in the coating. At the same time, the FTIR demonstrated spectra convergence involving broadening at the peak base from C=O stretching vibrations. Moreover, the coating’s colour change and hardness level were similar in both cases. An identical, cohesive failure was observed in the top coat in the adhesion test. Comparable values of the impedance module and phase angle were recorded. The extent of the coating change after the original cyclic test corresponds to two years of exposure on the test site, which was not observed after any other accelerated tests used in this study.

To summarize, the following can be stated:

- Accelerated weathering test methods established for solvent-based paints caused significantly different degradations of acrylic coatings than observed under natural conditions.
- An original cyclic weathering test for waterborne coatings was developed.
- After an original cyclic weathering test, degradations and changes were observed to be similar to that after two years of exposure on the test site for the following properties: surface morphology, colour, hardness, nature of failure and the impedance.

Considering the above, the programming direction of artificial weathering tests on waterborne coatings followed in this study can be considered adequate and prospective. There are plans to develop it in future studies. Modification of the original cyclic weathering by more prolonged exposure to UV radiation, humidity and reducing the SO_2_ concentration is being considered. A broader range of waterborne paints intended for steel structure corrosion protection, longer exposure time in natural conditions and variability and seasonality of various climate factors will also be taken into consideration.

## Figures and Tables

**Table 1:** Composition of acrylic paint.

Ingredient Name	%
titanium dioxide	≥10–≤25
trizinc bis(orthophosphate)	≥5–≤10
2-butoxyethanol	≥1–≤3
zinc oxide	≤0.9
trimethylolpropane	≤0.3
ammonia	≤0.3

**Table 2:** Acrylic paint characteristics.

Density (g/cm^3^)	Apparent Viscosity (Pa·s)	Content of Non-Volatile Ingredients (%)	Surface Drying Time (min)
1.308–1.339	0.69–0.71	61.2–61.8	30–35 (50 µm)

**Table 3:** Artificial weathering exposure.

Exposure Type	Water Condensation (H)				Neutral Salt Spray (NSS)			Original Cyclic Test (C)		
Exposure time, h	240	480	720	1000	360	720	1440	360 (1 cycle)	720 (2 cycles)	1080 (3 cycles)
Series marking	H_240_	H_480_	H_720_	H_1000_	NSS_360_	NSS_720_	NSS_1440_	C1	C2	C3

**Table 4:** A course of a single cycle in the original cyclic test (C).

Days 1–5 (120 h) UV—ISO 16474-3	Days 6–12 (168 h) NSS Test—ISO 9227	Day 13 (24 h) Freezing	Days 14–15 (2 × 24 h) Kesternich Test
One cycle included: - 4 h irradiation by lamp UVA-340 (0.83 W/m^2^, 60 ± 3 °C) - 4 h water condensation (50 ± 3 °C)	air temperature in the chamber: 35 ± 2 °C salt solution concentration: 5% NaCl (50 g NaCl/1 L distilled water)	temperature −20 ± 2 °C	One cycle included: - 1 L of sulphur dioxide - 8 h heating at 40 ± 3 °C, 100% RH - 16 h cooling with ventilation at 18–28 °C, <100% RH

**Table 5:** Results of macroscopic evaluation of the coating after weathering.

Series Designation	Blistering ISO 4628-2	Rusting ISO 4628-3	Cracking ISO 4628-4	Flaking ISO 4628-5	Chalking ISO 4628-6	Corrosion around a Scribe, mm ISO 4628-8
N4, N8, N12, N16, N20, N24	0S(0)	Ri0	0S(0)	0S(0)	0S(0)	0
H_240_, H_480_, H_7200_	0S(0)	Ri0	0S(0)	0S(0)	0S(0)	not evaluated
H_1000_	1S(1)	Ri0	0S(0)	0S(0)	0S(0)	not evaluated
NSS_360_	2(S1)/5(S2)	Ri3	0S(0)	0S(0)	0S(0)	0.5
NSS_720_	3(S2)	Ri4	0S(0)	0S(0)	0S(0)	0.5
NSS_1440_	5(S3)	Ri5	0S(0)	0S(0)	0S(0)	2.0
C1	0S(0)	Ri0	0S(0)	0S(0)	0S(0)	0
C2	0S(0)/4(S2)	Ri0	0S(0)	0S(0)	0S(0)	1.0
C3	0S(0)/4(S2)	Ri1	0S(0)	0S(0)	0S(0)	1.0

The results are expressed in degrees according to ISO 4628 [34] parts 2–6 and 8. The first component in the XS(Y) symbol, i.e., X stands for quantity, S means size and Y represents the size value (according to the description in item 2.4). If the same result was observed for all series in the group, it was presented once. The component after the slash (“/”) presents the results for incised samples but only when the result for the incised samples was different than that for non-incised ones.

**Table 6:** Coating TGA results.

No.	Sample	m_1_ (%)	m_2_ (%)	T_d5%_ (°C)	T_max_ (°C)	Res_750_ (%)
1	R	1.9	45.5	337	409	51.0
2	N_20_	1.8	43.7	341	409	52.7
3	N_24_	1.7	43.4	347	411	53.0
4	NSS_720_	1.7	43.4	354	409	53.0
5	C3	1.6	44.5	350	410	51.8

m_1_, m_2_—mass losses corresponding to coating dehydration and decomposition of the coating polymer matrix, respectively; T_d5%—_decomposition temperature corresponding to 5% mass loss; T_max_—temperature corresponding to the fastest decomposition kinetics, determined based on the derivative signal (dW/dT(%/°C)); Res_750_—pyrolysis residues determined for 750 °C.

**Table 7:** Summary of the determined glass transition temperature, melting point * and melting enthalpy *.

Test Series	Tg ^1^ (°C)	Tg ^2^ (°C)	Tm ^1^ (°C)	ΔH ^1^ (J/g)
R	19.2	17.0	117.2	29.5
N_20_	21.1	21.2	115.8	27.4
N_24_	21.8	22.5	113.7	25.3
NSS_720_	20.9	22.2	129.2	31.5
C3	23.3	23.6	137.1	20.5

* the transition is of an endothermal nature, but evaporation of water and/or 2-butoxyethanol is more likely than the melting of additive; indices ^1^ and ^2^ stand for the first and second heating cycle, respectively.

**Table 8:** ATR-FTIR absorption band positions.

Band/Material	R	N_20_	N_24_	NSS_720_	C3
* O-H stretching	3676 cm^−1^	3675 cm^−1^	3676 cm^−1^	3675 cm^−1^	3676 cm^−1^
C-H stretching	2956 cm^−1^ 2928 cm^−1^ 2873 cm^−1^	2956 cm^−1^ 2924 cm^−1^ 2872 cm^−1^	2957 cm^−1^ 2928 cm^−1^ 2873 cm^−1^	2956 cm^−1^ 2928 cm^−1^ 2872 cm^−1^	2956 cm^−1^ 2928 cm^−1^ 2872 cm^−1^
C=O stretching	1726 cm^−1^	1725 cm^−1^	1725 cm^−1^	1726 cm^−1^	1725 cm^−1^
C=C stretching	1603 cm^−1^ 1494 cm^−1^	1603 cm^−1^ 1494 cm^−1^	1603 cm^−1^ 1494 cm^−1^	1603 cm^−1^ 1494 cm^−1^	1603 cm^−1^ 1494 cm^−1^
C-H bending	1453 cm^−1^ 698 cm^−1^	1453 cm^−1^ 698 cm^−1^	1452 cm^−1^ 698 cm^−1^	1453 cm^−1^ 698 cm^−1^	1453 cm^−1^ 698 cm^−1^
C-O stretching	1158 cm^−1^	1159 cm^−1^	1158 cm^−1^	1158 cm^−1^	1158 cm^−1^
C-H rock	840 cm^−1^	831 cm^−1^	830 cm^−1^	840 cm^−1^	836 cm^−1^
* Si-O stretching	1014 cm^−1^ 665 cm^−1^	1008 cm^−1^ 665 cm^−1^	1011 cm^−1^ 666 cm^−1^	1011 cm^−1^ 665 cm^−1^	1015 cm^−1^ 666 cm^−1^

* bands characteristic of inorganic filler.

## Data Availability

The data presented in this study are available on request from the corresponding author (due to privacy).

## References

[1] Nash W., Zheng L., Birbilis N. (2022). Deep Learning Corrosion Detection with Confidence. Npj Mater. Degrad..

[2] Yin B., Considine T.A., Emdad F., Kelley J.V., Jensen R.E., Rundensteiner E.A. Corrosion Assessment: Data Mining for Quantifying Associations between Indoor Accelerated and Outdoor Natural Tests. Proceedings of the 2020 IEEE International Conference on Big Data (Big Data).

[3] Wan Mohamad Kamaruzzaman W.M.I., Shaifudin M.S., Mohd Nasir N.A., Syafiq Mohd Hamidi N.A., Yusof N., Adnan A., Jew L.O., Norsani Wan Nik W.M., Mohd Ghazali M.S. (2022). Eggshells Biowaste Filler for Improving the Anticorrosive Behaviour of Waterborne Polyurethane Coatings on Mild Steel in Artificial Seawater. J. Mater. Res. Technol..

[4] Stojanović I., Cindrić I., Turkalj L., Kurtela M., Rakela-Ristevski D. (2022). Durability and Corrosion Properties of Waterborne Coating Systems on Mild Steel Dried under Atmospheric Conditions and by Infrared Radiation. Materials.

[5] Ecco L.G., Fedel M., Deflorian F., Becker J., Iversen B.B., Mamakhel A. (2016). Waterborne Acrylic Paint System Based on Nanoceria for Corrosion Protection of Steel. Prog. Org. Coat..

[6] Dhar M., Mishra C., Das A., Manna U. (2024). Polymerization of Monomer Aggregates for Tailoring and Patterning Water Wettability. Chem. Commun..

[7] Roselli S.N., Romagnoli R., Deyá C. (2017). The Anti-Corrosion Performance of Water-Borne Paints in Long Term Tests. Prog. Org. Coat..

[8] Nguyen T.V., Le X.H., Dao P.H., Decker C., Nguyen-Tri P. (2018). Stability of Acrylic Polyurethane Coatings under Accelerated Aging Tests and Natural Outdoor Exposure: The Critical Role of the Used Photo-Stabilizers. Prog. Org. Coat..

[9] Rodrigues Peruchi A.B., Zuchinali F.F., Bernardin A.M. (2021). Development of a Water-Based Acrylic Paint with Resistance to Efflorescence and Test Method to Determine the Appearance of Stains. J. Build. Eng..

[10] Kumar S., Dhar M., Prusty B.M., Sarkar D., Das A., Manna D., Manna U. (2023). Amidation Reaction to Derive Waterborne, Tolerant, and Optically Transparent Solid Slippery and Superhydrophobic Coatings. Chem. Eng. J..

[11] Nguyen T.V., Dao P.H., Nguyen T.A., Dang V.H., Ha M.N., Nguyen T.T.T., Vu Q.T., Nguyen N.L., Dang T.C., Nguyen-Tri P. (2020). Photocatalytic Degradation and Heat Reflectance Recovery of Waterborne Acrylic Polymer/ZnO Nanocomposite Coating. J. Appl. Polym. Sci..

[12] Zhou Z., Meng Q., Wang J., Ren P., Li C., Wang Z., Tan H. (2024). Impact of Marine Atmospheric Corrosion on the Thermophysical Properties of Building Coatings. J. Build. Eng..

[13] Meng Y., Gao Y., Li J., Liu J., Wang X., Yu F., Wang T., Gao K., Zhang Z. (2022). Preparation and Characterization of Cross-Linked Waterborne Acrylic/PTFE Composite Coating with Good Hydrophobicity and Anticorrosion Properties. Colloids Surf. A Physicochem. Eng. Asp..

[14] Liu M., Mao X., Zhu H., Lin A., Wang D. (2013). Water and Corrosion Resistance of Epoxy-Acrylic-Amine Waterborne Coatings: Effects of Resin Molecular Weight, Polar Group and Hydrophobic Segment. Corros. Sci..

[15] Pagnin L., Calvini R., Wiesinger R., Schreiner M. (2021). SO_2−_ and NO_x−_ Initiated Atmospheric Degradation of Polymeric Films: Morphological and Chemical Changes, Influence of Relative Humidity and Inorganic Pigments. Microchem. J..

[16] Cogulet A., Blanchet P., Landry V. (2019). Evaluation of the Impacts of Four Weathering Methods on Two Acrylic Paints: Showcasing Distinctions and Particularities. Coatings.

[17] Deflorian F., Rossi S., Fedel M. (2008). Organic Coatings Degradation: Comparison between Natural and Artificial Weathering. Corros. Sci..

[18] Fedel M., Rossi S., Deflorian F. (2013). Comparison between Natural and Artificial Weathering of E-Coated Galvanized Steel Panels. Prog. Org. Coat..

[19] Scrinzi E., Rossi S., Orian F.D. (2011). Influence of Natural and Artifi Cial Weathering on Aesthetic and Protective Properties of Organic Coatings. Corros. Rev..

[20] Jacques L.F.E. (2000). Accelerated and Outdoor/Natural Exposure Testing of Coatings. Prog. Polym. Sci..

[21] Davalos-Monteiro R., D’Ambrosio G., Zhou X., Gibbon S., Curioni M. (2021). Relationship between Natural Exposure Testing and Cyclic Corrosion Testing ISO 20340 for the Assessment of the Durability of Powder-Coated Steel. Corros. Eng. Sci. Technol..

[22] Pélissier K., Le Bozec N., Thierry D., Larché N. (2022). Evaluation of the Long-Term Performance of Marine and Offshore Coatings System Exposed on a Traditional Stationary Site and an Operating Ship and Its Correlation to Accelerated Test. Coatings.

[23] Ramdé T., Ecco L.G., Rossi S. (2017). Visual Appearance Durability as Function of Natural and Accelerated Ageing of Electrophoretic Styrene-Acrylic Coatings: Influence of Yellow Pigment Concentration. Prog. Org. Coat..

[24] Valverde J.C., Moya R. (2014). Correlation and Modeling between Color Variation and Quality of the Surface between Accelerated and Natural Tropical Weathering in Acacia Mangium, Cedrela Odorata and Tectona Grandis Wood with Two Coating. Color Res. Appl..

[25] Cristoforetti A., Rossi S., Deflorian F., Fedel M. (2023). Comparative Study between Natural and Artificial Weathering of Acrylic-Coated Steel, Aluminum, and Galvanized Steel. Mater. Corros..

[26] Shang B., Zhang L., Zhu Y., Wu S., Teng J., He Q., Su Y. (2021). Corrosion Degradation of Two Coating Systems Exposed for Three Years in a Tropical Oceanic Atmospheric Environment. Int. J. Electrochem. Sci..

[27] Deflorian F., Rossi S., Fedrizzi L., Zanella C. (2007). Comparison of Organic Coating Accelerated Tests and Natural Weathering Considering Meteorological Data. Prog. Org. Coat..

[28] (2007). Preparation of Steel Substrates before Application of Paints and Related Products Visual Assessment of Surface Cleanliness. Part 1: Rust Grades and Preparation Grades of Uncoated Steel Substrates and of Steel Substrates after Overall Removal of Previous Coatings.

[29] (2017). Paints and Varnishes Corrosion Protection of Steel Structures by Protective Paint Systems—Part 2: Classification of Environments.

[30] (2017). Paints and Varnishes Determination of Resistance to Humidity—Part 1: Condensation (Single-Sided Exposure).

[31] (2017). Corrosion Tests in Artificial Atmospheres Salt Spray Tests.

[32] (2021). Paints and Varnishes Methods of Exposure to Laboratory Light Sources—Part 3: Fluorescent UV Lamps.

[33] (1993). Paints and Varnishes Determination of Resistance to Humid Atmospheres Containing Sulfur Dioxide.

[34] (2016). Paints and Varnishes Evaluation of Degradation of Coatings—Designation of Quantity and Size of Defects, and Intensity of Uniform Changes in Appearance.

[35] (2016). Paints and Varnishes Pull-Off Test for Adhesion.

[36] (2003). Paints and Varnishes Buchholz Indentation Test.

[37] Effendy S., Zhou T., Eichman H., Petr M., Bazant M.Z. (2021). Blistering Failure of Elastic Coatings with Applications to Corrosion Resistance. Soft Matter.

[38] Gao J., Li C., Lv Z., Wang R., Wu D., Li X. (2019). Correlation between the Surface Aging of Acrylic Polyurethane Coatings and Environmental Factors. Prog. Org. Coat..

[39] Bauer F., Decker U., Naumov S., Riedel C. (2014). Photoinitiator-Free UV Curing and Matting of Acrylate-Based Nanocomposite Coatings: Part 3. Prog. Org. Coat..

[40] Kotnarowska D., Stanisławek D. (2018). Influence of Climatic Ageing on Acrylic Coatings Adhesion. Autobusy.

[41] Hurley C.J., Zahavi J., Schmitt G.F. (1983). UV Radiation Exposure Effects on the Rain Erosion Resistance of Coated Monolithic Poly(Carbonate) Transparencies. Wear.

[42] Schulz U., Trubiroha P., Schernau U., Baumgart H. (2000). The Effects of Acid Rain on the Appearance of Automotive Paint Systems Studied Outdoors and in a New Artificial Weathering Test. Prog. Org. Coat..

[43] Pan T., Yu Q. (2016). Long-Term Anti-Corrosion Performance of a Conducting Polymer-Based Coating System for Steels. J. Mater. Eng. Perform..

[44] Fyfe C.A., McKinnon M.S. (1986). Investigation of the Thermal Degradation of Poly(Acrylic Acid) and Poly(Methacrylic Acid) by High-Resolution Carbon-13 CP/MAS NMR Spectroscopy. Macromolecules.

[45] Gohari G., Mohammadi A., Akbari A., Panahirad S., Dadpour M.R., Fotopoulos V., Kimura S. (2020). Titanium Dioxide Nanoparticles (TiO2 NPs) Promote Growth and Ameliorate Salinity Stress Effects on Essential Oil Profile and Biochemical Attributes of Dracocephalum Moldavica. Sci. Rep..

[46] Guzmán-Aponte L., Mejia R., Maury-Ramirez A. (2017). Metakaolin-Based Geopolymer with Added TiO 2 Particles: Physicomechanical Characteristics. Coatings.

[47] Mai Q., Zhou H., Ou L. (2021). Flotation Separation of Chalcopyrite and Talc Using Calcium Ions and Calcium Lignosulfonate as a Combined Depressant. Metals.

[48] Shameli K., Ahmad M.B., Yunus W.Z.W., Ibrahim N.A., Darroudi M. (2010). Synthesis and Characterization of Silver/Talc Nanocomposites Using the Wet Chemical Reduction Method. Int. J. Nanomed..

[49] Djangang C.N., Mbey J.A., Ekani C.J., Tiam S.T., Blanchart P., Njopwouo D. (2020). Improved Microstructure and Free Efflorescence Geopolymer Binders. SN Appl. Sci..

[50] Corona J.C., Jenkins D.M., Dyar M.D. (2015). The Experimental Incorporation of Fe into Talc: A Study Using X-Ray Diffraction, Fourier Transform Infrared Spectroscopy, and Mössbauer Spectroscopy. Contrib. Mineral. Petrol..

[51] Pintus V., Wei S., Schreiner M. (2016). Accelerated UV Ageing Studies of Acrylic, Alkyd, and Polyvinyl Acetate Paints: Influence of Inorganic Pigments. Microchem. J..

[52] Özgenç Ö., Durmaz S., Şahin S., Boyaci İ.H. (2020). Evaluation of the Weathering Resistance of Waterborne Acrylic- and Alkyd-Based Coatings Containing HALS, UV Absorber, and Bark Extracts on Wood Surfaces. J. Coat. Technol. Res..

[53] Kotnarowska D. (2010). Epoxy Coating Destruction as a Result of Sulphuric Acid Aqueous Solution Action. Prog. Org. Coat..

[54] Sun W., Xing C., Tang X., Zuo Y., Tang Y., Zhao X. (2021). Comparative Study on the Degradation of a Zinc-Rich Epoxy Primer/Acrylic Polyurethane Coating in Different Simulated Atmospheric Solutions. J. Coat. Technol. Res..

[55] Zhang H., Dun Y., Tang Y., Zuo Y., Zhao X. (2016). Correlation between Natural Exposure and Artificial Ageing Test for Typical Marine Coating Systems. J. Appl. Polym. Sci..

[56] Shao V., Wang X., Xing W., Zhang G. Correlation Analysis of Natural Environment Test and Laboratory Accelerated Test. Proceedings of the 2017 Second International Conference on Reliability Systems Engineering (ICRSE).

